# Evaluating the translation of implementation science to clinical artificial intelligence: a bibliometric study of qualitative research

**DOI:** 10.3389/frhs.2023.1161822

**Published:** 2023-07-10

**Authors:** H. D. J. Hogg, M. Al-Zubaidy, P. A. Keane, G. Hughes, F. R. Beyer, G. Maniatopoulos

**Affiliations:** ^1^ Faculty of Medical Sciences, Newcastle University, Newcastle Upon Tyne, United Kingdom; ^2^ The Royal Victoria Infirmary, Newcastle Upon Tyne Hospitals NHS Foundation Trust, Newcastle Upon Tyne, United Kingdom; ^3^ Moorfields Eye Hospital NHS Foundation Trust, London, United Kingdom; ^4^ Institute of Ophthalmology, University College London, London, United Kingdom; ^5^ Nuffield Department of Primary Care Health Sciences, Oxford University, Oxford, United Kingdom; ^6^ University of Leicester School of Business, University of Leicester, Leicester, United Kingdom; ^7^ Evidence Synthesis Group, Population Health Sciences Institute, Newcastle University, Newcastle Upon Tyne, United Kingdom

**Keywords:** artificial intelligence, clinical decision support tools, implementation, qualitative research, theory, theoretical approach, bibliometric study

## Abstract

**Introduction:**

Whilst a theoretical basis for implementation research is seen as advantageous, there is little clarity over if and how the application of theories, models or frameworks (TMF) impact implementation outcomes. Clinical artificial intelligence (AI) continues to receive multi-stakeholder interest and investment, yet a significant implementation gap remains. This bibliometric study aims to measure and characterize TMF application in qualitative clinical AI research to identify opportunities to improve research practice and its impact on clinical AI implementation.

**Methods:**

Qualitative research of stakeholder perspectives on clinical AI published between January 2014 and October 2022 was systematically identified. Eligible studies were characterized by their publication type, clinical and geographical context, type of clinical AI studied, data collection method, participants and application of any TMF. Each TMF applied by eligible studies, its justification and mode of application was characterized.

**Results:**

Of 202 eligible studies, 70 (34.7%) applied a TMF. There was an 8-fold increase in the number of publications between 2014 and 2022 but no significant increase in the proportion applying TMFs. Of the 50 TMFs applied, 40 (80%) were only applied once, with the Technology Acceptance Model applied most frequently (*n* = 9). Seven TMFs were novel contributions embedded within an eligible study. A minority of studies justified TMF application (*n* = 51,58.6%) and it was uncommon to discuss an alternative TMF or the limitations of the one selected (*n* = 11,12.6%). The most common way in which a TMF was applied in eligible studies was data analysis (*n* = 44,50.6%). Implementation guidelines or tools were explicitly referenced by 2 reports (1.0%).

**Conclusion:**

TMFs have not been commonly applied in qualitative research of clinical AI. When TMFs have been applied there has been (i) little consensus on TMF selection (ii) limited description of selection rationale and (iii) lack of clarity over how TMFs inform research. We consider this to represent an opportunity to improve implementation science's translation to clinical AI research and clinical AI into practice by promoting the rigor and frequency of TMF application. We recommend that the finite resources of the implementation science community are diverted toward increasing accessibility and engagement with theory informed practices. The considered application of theories, models and frameworks (TMF) are thought to contribute to the impact of implementation science on the translation of innovations into real-world care. The frequency and nature of TMF use are yet to be described within digital health innovations, including the prominent field of clinical AI. A well-known implementation gap, coined as the “AI chasm” continues to limit the impact of clinical AI on real-world care. From this bibliometric study of the frequency and quality of TMF use within qualitative clinical AI research, we found that TMFs are usually not applied, their selection is highly varied between studies and there is not often a convincing rationale for their selection. Promoting the rigor and frequency of TMF use appears to present an opportunity to improve the translation of clinical AI into practice.

## 1. Introduction

Implementation science is a relatively young field drawing on diverse epistemological approaches and disciplines across a spectrum of research and practice (1). Its pragmatic goal of bridging know-do gaps to improve real-world healthcare necessitates this multi-disciplinary approach (2). A key aspect of implementation science is the application of theories, models or frameworks (TMF) to inform or explain implementation processes and determinants in a particular healthcare context (2, 3). In recent years TMFs addressing the implementation of interventions in healthcare organisations have accelerated and are pursued across a large and diverse literature which seeks to explore the factors shaping the implementation process (4). In line with the applications of TMFs, implementation researchers have variously employed qualitative research to explore the dynamic context and systems into which evidence-based interventions are embedded into practice by addressing the “hows and whys” of implementation (5). Drawing upon distinctive theoretical foundations, qualitative methodologies have offered a range of different analytical lenses to explore the complex processes and interactions shaping implementation through the recursive relationship between human action and the wider organisational and system context (4). Although this diversity of approach has allowed researchers to align specific research questions and objectives with particular context(s) at the policy, systems and organisational levels, at the same time it may pose challenges in informing the selection criteria for researchers to choose from the many TMFs in the field (6). This risks perpetuating or expanding implementation researchers' disconnect with practitioners, on whom implementation science's goal of improving real-world healthcare depends (7).

Healthcare interventions centering on clinical artificial intelligence (AI) appear in particular need of the proposed benefits of implementation science, as they are subject to a persistent know-do gap coined the “AI chasm” (8). Computer-based AI was conceived more than 50 years ago and has been incorporated into clinical practice through computerized decision support tools for several decades (9, 10). However, advancing computational capacity and the feasibility and potential of deep learning methods have galvanized public and professional enthusiasm for all applications of AI, including healthcare (11). The acknowledgment of this potential is formalized in the embedment of clinical AI into national healthcare strategic plans and by the recent surge of regulatory approvals issued for “software/AI as a medical device” (12–14). Despite this, there are few examples of clinical AI implemented in real-world patient care and little evidence of the benefits it has brought about (15, 16). This is in part because of the sensitivity of clinical AI interventions to technical, social and organizational variations in the context into which they are implemented and the paucity of research insights that go beyond the efficacy or effectiveness of the interventions themselves (17). TMFs offer a potential solution to this challenge as they allow insights from specific interventions and contexts to be abstracted to a degree through which they remain actionable whilst becoming transferrable across a wider range of interventions and contexts (18).

It is outside of the scope of the present study to directly assess the impact of implementation science on the translation of clinical AI to practice due to the bias and scarcity of reports of implementation success or failure (19). However, having been consistently proposed as an indicator of high-quality implementation research, the frequency and nature of TMF application to clinical AI research seem likely to influence the speed and extent of clinical AI interventions' real-world impact. To establish how the application of TMFs can most effectively support the realization of patient benefit from clinical AI, it will first be necessary to understand how they are currently applied. Given the early translational stage of most clinical AI research and the relatively low number of interventions that have been implemented to date, it seems unlikely that implementation science principals such as TMF usage are as well established as they are for other healthcare interventions. Implementation research focused on other categories of healthcare interventions has been characterized through descriptive summaries of TMF selection and usage. These studies act as a frame of reference, but to our knowledge none report on digital health interventions (20–22).

This bibliometric study aims to measure and characterize the application of TMFs in qualitative clinical AI research. These data are intended to (i) identify TMFs applied in contemporary clinical AI research, (ii) provide insight into implementation research practices in clinical AI and (iii) inform strategies which may improve the efficacy of implementation science in clinical AI research.

## 2. Methods

**Figure 1:**
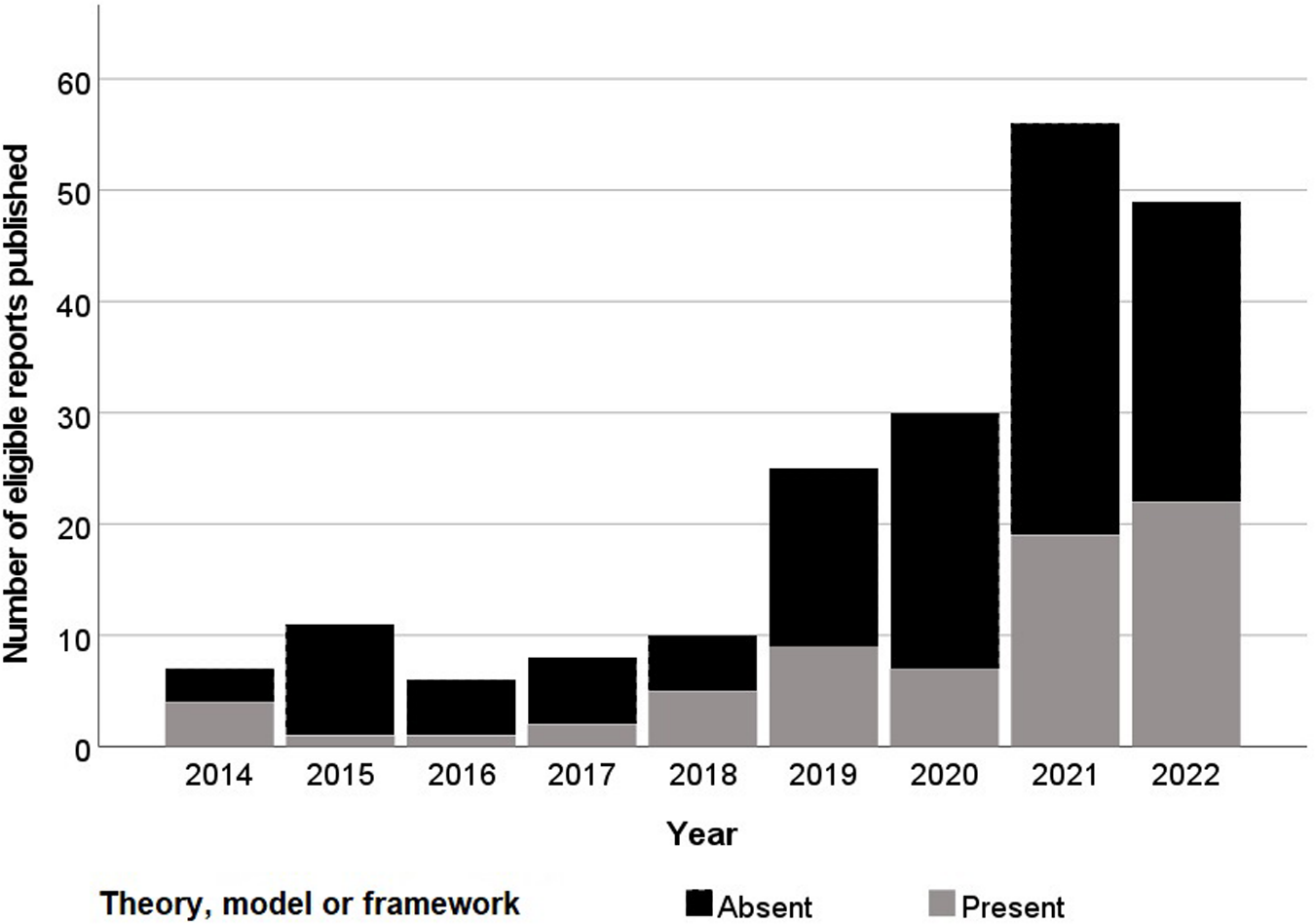
Histogram of year of publication of eligible reports and their application of a theory, model or framework.

Mobilising a definition of implementation research, e.g., research “focused on the adoption or uptake of clinical interventions by providers and/or systems of care”, for a systematic search strategy is challenged by variation in approaches to article indexing and the framing which researchers from varied disciplines lend to their work (23–25). The present study aimed to mitigate this by targeting primary qualitative research of clinical AI. Qualitative research has a foundational relationship with the application of TMFs in implementation science and its focus on understanding how implementation processes shape and are shaped by dynamic contextual factors. Developing such an understanding requires an exploration of human behaviours, perceptions, experiences, attitudes and interactions. This approach was intended to maximise the sensitivity with which clinical AI implementation research using TMFs was identified whilst maintaining a feasible specificity of the search strategy (Figure 1).

This bibliometric study updates a pre-existent search strategy using AND logic to combine qualitative research with two other concepts; AI-enabled decision support including rule-based and non-rule-based tools and any healthcare context (17, 27). The earliest eligibility date of January 2014 was maintained from this prior work, marking the first FDA approvals for “Software as a Medical Device” (13), but the updated search execution included studies published up to October 2022. The five original target databases were maintained; Scopus, CINAHL (EBSCO), ACM Digital Library and Science Citation Index (Web of Science) to cover computer science, allied health, medical and grey literature (Supplementary File S1). Only English language indexing was required, there were no exclusion criteria relating to full-text language. The initial results were de-duplicated using Endnote x9.3.3 (Clarivate Analytics, PA, USA) and two independent reviewers (HDJH, MA) performed full title and abstract screening using Rayyan (28). The process was overseen by an information specialist (FB) and screening disagreements were arbitrated by a separate senior implementation researcher (GM). Eligible review and protocol manuscripts were included for reference hand searching only. Full-text review was performed independently by two independent reviewers (HDJH, MA), with the same arbiter (GM).

Two reviewers (HDJH, MA) extracted characteristics from articles independently following an initial consensus exercise. These characteristics included the year and type of publication, source field and impact factor, implementation context studied, TMF application, study methods and study participant type and number. For each study referring to a TMF in the body text, the stage of the research at which it had contributed and any justification for its selection was noted. The index article for the TMFs applied in eligible reports were sourced to facilitate characterization by a single reviewer (HDJH) following consensus exercises with a senior implementation researcher (GM). Nilsen's 5-part taxonomy of TMF types (process models, determinant frameworks, classic theories, implementation theories and evaluation frameworks) and Liberati's taxonomy of TMFs' disciplinary roots (usability, technology acceptance, organizational theories and practice theories) were applied to characterize each TMF along with its year of publication (29, 30).

## 3. Results

### 3.1. Eligible study characteristics

**Figure 2:**
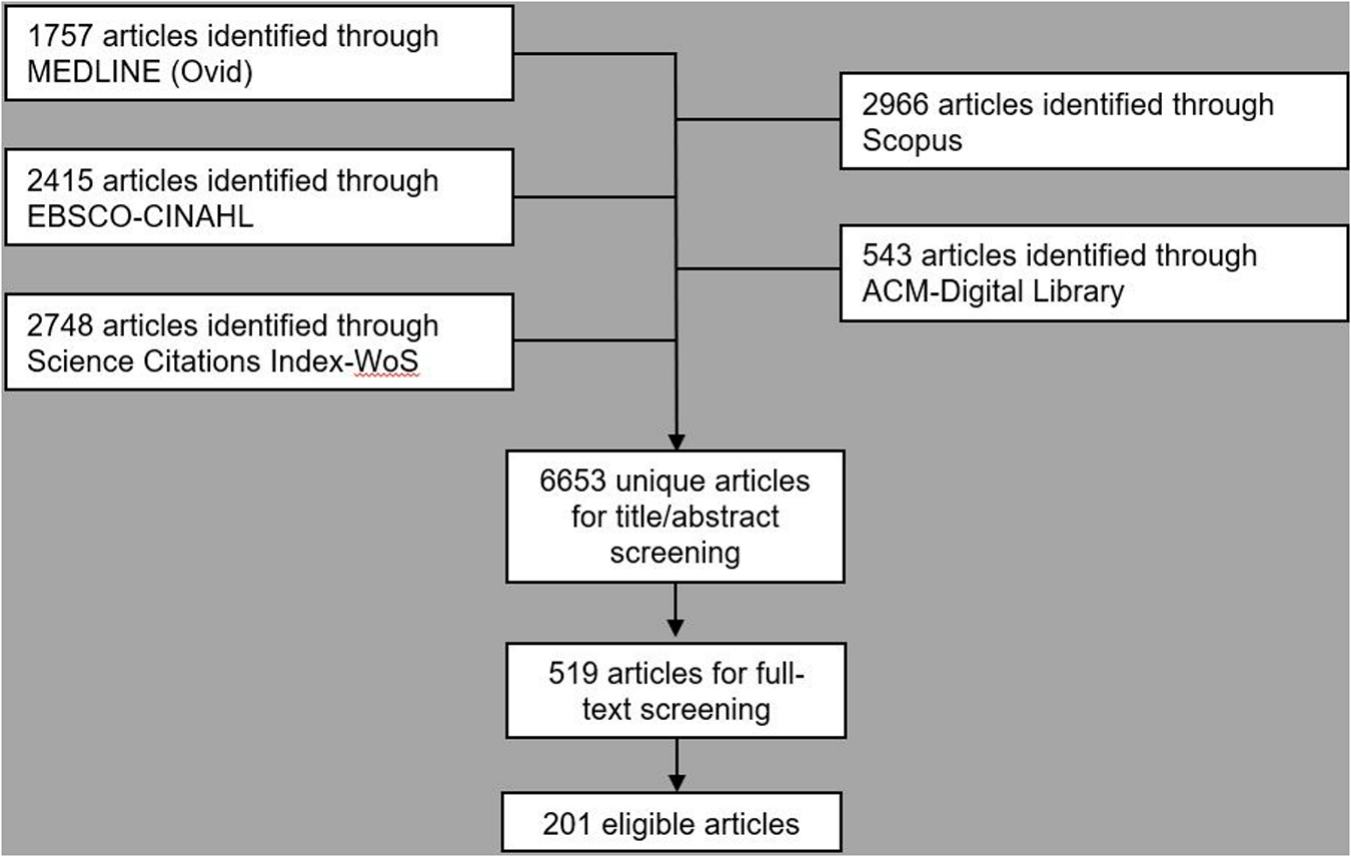
PRISMA style flowchart of database searching, de-duplication and title, abstract and full-text screening (26).

Following initial deduplication 6,653 potential eligible titles were returned by searches, 519 (7.8%) of which were included following title and abstract screening. Full-text screening identified 202 unique eligible studies (Figure 1). Three (1.5%) of these reports were theses with the remaining 198 (98.5%) consisting of articles in academic journals (Table 1). Excluding 2016, the frequency of eligible publication increased year-on-year, with a monthly rate of 4.9 publications averaged over January-October 2022 compared to 0.6 between January-December 2014 (Figure 2). Thirty-five different countries hosted the healthcare context under study, with the United States (*n* = 56, 27.7%), United Kingdom (*n* = 29, 14.4%), Canada (*n* = 16, 8.0%), Australia (*n* = 16, 7.9%) and Germany (*n* = 11, 5.4%) the most frequent countries studied. Six studies (3.0%) were based in countries categorized by the United Nations as having a medium or low human development index (31). Of the 172 studies focused on a single clinical specialty, primary care (*n* = 48, 27.9%) and psychiatry (*n* = 16, 9.3%) were the most common of 27 distinct clinical specialties.

**Table 1:** Characteristics of 202 eligible reports.

Characteristic	Category	Number of reports (%)
Scope of source	Clinical	90 (44.6%)
	Health service management	91 (45.0%)
	Health informatics	16 (7.9%)
	Other	5 (2.5%)
Context of AI application studied	Hypothetical	78 (38.6%)
	Simulated	46 (22.8%)
	Clinical	78 (38.6%)
AI type studied	Not specified	16 (7.9%)
	Rule-based	88 (43.6%)
	Machine learning	98 (48.5%)
Data collection method	Interviews	105 (52.0%)
	Focus groups	34 (16.8%)
	Survey	24 (11.9%)
	Observation	3 (1.5%)
	Mixed	36 (17.8%)
Participants	Clinicians	105 (52.0%)
	Patients and the public	26 (12.9%)
	Managers and leaders	2 (1.0%)
	Developers	2 (1.0%)
	Policy makers and	2 (1.0%)
	Mixed	65 (32.2%)

### 3.2. Theory, model or framework characteristics

Seventy eligible reports (34.7%) applied at least one of 50 distinct TMFs in the main text (Table 2), 7 (14.0%) of these were new TMFs developed within the eligible article itself. Theory application was increasingly prevalent as studies focused closer toward real-world use, with studies of hypothetical, simulated or active clinical use cases applying TMFs in 26.9%, 34.8% and 42.3% of studies respectively. There was no significant difference between the frequency of TMF application before and after the start of 2021, the median year of publication (Chi squared test, *p* = 0.17). Twelve (17.1%) of the 70 reports drawing on a TMF applied more than one [maximum 5 (82)]. Of the 87 instances that a TMF was applied it originated from the fields of technology acceptance (*n* = 36, 41.4%), practice theory (*n* = 21, 24.1%), organizational theory (*n* = 19, 21.8%) or usability (*n* = 11, 12.6%) according to Liberati's taxonomy (30). Similarly, under Nilsen's taxonomy of TMFs the purpose of each TMF applied could be classified as determinant framework (*n* = 49, 56.3%), process model (*n* = 18, 20.7%), classic theory (*n* = 10, 11.5%), evaluation framework (*n* = 9, 10.3%) or implementation theory (*n* = 1, 1.1%) (29).

**Table 2:** Theories, models and frameworks applied by eligible reports.

Theory, model or framework	Year of index publication	Liberati classification (30)	Nilsen classification (29)	Frequency of use
Awareness-to-Adherence Model (32)	1996	Practice theory	Process model	1
Behaviour change technique taxonomy (33)	2013	Technology acceptance	Evaluation framework	1
Behaviour change theory (34)	1977	Technology acceptance	Classic theory	1
Behaviour change wheel (35)	2011	Technology acceptance	Determinant framework	5
Biography of Artefact (36)	2010	Practice theory	Classic theory	1
Consolidated Framework for Implementation Research (37)	2009	Organizational theory	Determinant framework	7
Clinical adoption meta-model (38)	2014	Technology acceptance	Evaluation framework	1
Clinical performance feedback intervention theory (39)	2019	Technology acceptance	Determinant framework	1
Disruptive innovation theory (40)	1995	Organizational theory	Classic theory	1
Dual process model of reasoning (41)	2009	Technology acceptance	Classic theory	1
Expectancy-value theory (42)	2000	Technology acceptance	Classic theory	1
Fit Between Individuals Task and Technology (43)	2006	Technology acceptance	Evaluation framework	1
Flottorp framework (44)	2013	Practice theory	Determinant framework	1
Framework for designing user-centred displays of explanation (45)	2020	Usability	Determinant framework	2
Framework of patient orientation to applications of AI in healthcare (46)	2022	Practice theory	Process model	1
Goal directed design (47)	1995	Usability	Process model	1
Heuristic evaluation (48)	1990	Usability	Determinant framework	2
Human-computer trust conceptual framework (49)	2000	Usability	Process model	1
Innovation-decision process framework (50)	2013	Organizational theory	Classic theory	1
Intention to use AI Model (51)	2020	Technology acceptance	Determinant framework	1
Iterative, collaborative development and implementation framework (52)	2021	Organizational theory	Process model	1
Kano model of satisfaction (53)	1984	Usability	Determinant framework	1
Methontology (54)	1997	Usability	Process model	1
Machine learning maturity model (55)	2021	Technology acceptance	Determinant framework	1
GPs’ determinants of attitude towards AI-enabled systems (56)	2022	Technology acceptance	Process model	1
Non-adoption, Abandonment, Scale-up, Spread and Sustainability (57)	2017	Organizational theory	Determinant framework	2
Normalisation process model (58)	2007	Practice theory	Process model	1
Normalisation process theory (59)	2009	Practice theory	Mixed	4
Occupational therapy intervention process model (60)	1998	Practice theory	Process model	1
PESTLE framework (61)	1967	Organizational theory	Evaluation framework	1
Positions of perceived control (62)	2015	Practice theory	Evaluation framework	1
Process-oriented model of implementation pathways (63)	2020	Technology acceptance	Process model	1
Programme sustainability assessment tool (64)	2014	Practice theory	Determinant framework	1
Rasmussen behaviour model (65)	1983	Usability	Classic theory	1
Rogers’ Theory of Diffusion (66)	1962	Practice theory	Classic theory	1
Shackel model (67)	1991	Usability	Determinant framework	1
Sittig and Singh sociotechnical framework (68)	2010	Practice theory	Determinant framework	6
Strong structuration theory (69)	2007	Organizational theory	Determinant framework	1
Systems engineering for patient safety 3.0 (70)	2020	Organizational theory	Determinant framework	1
Systems-Theoretic Accident and Process Analysis (71)	2011	Organizational theory	Evaluation framework	1
Technology acceptance model (72)	1989	Technology acceptance	Determinant framework	9
Theoretical domains framework (73)	2005	Technology acceptance	Mixed	3
Theoretical framing theory (74)	1999	Organizational theory	Classic theory	1
Theory of meaningful human control (75)	2018	Practice theory	Classic theory	1
Theory of planned behavior (76)	1991	Technology acceptance	Determinant framework	1
Two component model of attitude (77)	1961	Technology acceptance	Process model	1
Unified Theory of Acceptance and Use of Technology (78)	2003	Technology acceptance	Determinant framework	7
Usabilty criteria of Scapin and Bastien (79)	1997	Usability	Determinant framework	1
User-driven co-development of AI model (80)	2021	Practice theory	Process model	1
Work as done (81)	2015	Organizational theory	Classic theory	1

AI, artificial intelligence; GP, general practitioners; PESTLE, political, economic, sociological, technological, legal and environmental.

### 3.3. Justification and application of theories, models and frameworks

The Technology Acceptance Model was the most frequent choice when a TMF was applied (*n* = 9, 12.9%), but 40 (80.0%) of the TMFs were only applied once across all eligible reports. Across the 87 instances of reports explicitly applying a TMF, 4 different modes of application emerged; to inform the study or intervention design (*n* = 9, 10.3%), to inform data collection (*n* = 29, 33.3%), to inform data analysis (*n* = 44, 50.6%) and to relate or disseminate findings to the literature (*n* = 25, 28.7%). The majority of instances in which a report applied a TMF carried no explanation or justification (*n* = 51, 58.6%). Five (5.7%) reports made isolated endorsement of the TMF's popularity or quality, e.g., “The sociotechnical approach has been applied widely…” (83). Thirty-one (35.6%) outlined the alignment of the TMF and the present research question, e.g., “our findings are consistent with disruptive innovation theory…” (84). Eleven (12.6%) reports discussed the disadvantages and alternatives that had been considered, e.g., “Because this model does not consider the unique characteristics of the clinical setting… we further adopted qualitative research techniques based on the CFIR [Consolidated Framework for Implementation Research] to further identify barriers and facilitators of the AI-based CDSS [Clinical Decision Support System]” (85).

## 4. Discussion

### 4.1. Principal findings

This study shows that a minority of clinical AI qualitative research applies TMFs, with no suggestion of a change in the relative frequency of TMF application over time. This appears to contrast with research funders and policy makers increasingly valuing more theory-based definitions of evidence and the consistent requirement for TMFs in related reporting guidelines and evaluation criteria (25, 86–88). Underlying this increasing appreciation of the contribution that TMFs can make, is a perception that specific research questions with unique configurations of complexity can draw on prior knowledge through the application of a well-matched theoretical approach (29). It is the great variety of unique research questions that may justify the ever-increasing variety of available TMFs. If considered matching of a specific research question's demands and a specific TMF's value is not taking place however, the ongoing proliferation of TMFs may only serve to further alienate practitioners trying to make sense of the shifting landscape of TMFs (7).

Within this study's relatively narrow eligibility criterion of qualitative clinical AI research, the variety and inconsistency of TMFs applied was striking, with 80% of the 50 TMFs encountered only applied once. This variation in TMF selection was also mirrored by the their varied purpose and mode of application. Across these applications of TMFs, a convincing rationale for their selection was usually absent. This heterogenous TMF selection coupled with little evidence of considered selection, suggests that current TMF application in qualitative clinical AI research usually fails to satisfy established definitions of good practice in implementation research (2, 25). If it is assumed that meeting these definitions of good practice would more effectively support implementation science's goal of bridging know-do-gaps, then it seems likely TMF application is currently under-delivering for efforts to translate clinical AI into practice. The observed heterogeneity in TMF selection is also set to grow, as 15% of the theories applied in eligible articles were novel. This may improve current practice in TMF application if these novel TMFs better serve the needs of research questions in clinical AI implementation. However, only 1 of these 7 novel TMFs has been applied within the other eligible reports of this bibliometric study and so there is a real risk of exacerbating unjustified heterogeneity in TMF usage (45).

### 4.2. Comparison with prior work

To the best of our knowledge, there are no other reviews of TMF application in qualitative implementation research of digital health. Smaller scoping reviews concerning specific disease areas and clinical guideline implementation, and a survey of implementation scientist practices are published, but their findings differ to the present study's in two important regards. Firstly, the heterogeneity of TMF application selection appears to be much greater in the present study, with half of guideline implementation studies applying at least one of the same 2 TMFs (20, 21). The preferences across implementation scientists in general also seem to differ from researchers working on clinical AI implementation as only 2 of the TMFs identified in the present study (Theoretical Domains Framework and Consolidated Framework for Implementation Research) appeared in the 10 most frequently applied TMFs from a survey of an international cohort of 223 implementation scientists (6). These differing preferences may be accounted for by the prominence of TMFs in qualitative clinical AI research from Technology Acceptance disciplines (40.9%), as described by Liberati's taxonomy, which do not have such natural relevance across implementation science as a whole (30). Secondly, the frequency with which any degree of rationale for TMF selection was described in the present study (42%) appears much lower than the 83% observed in guideline implementation research (21). Both of these differences seem to reflect the field of clinical AI and its nascent engagement with formally trained implementation scientists who have more established means of selecting TMFs (6). Taken together, the heterogenous and unjustified selection of TMFs suggests superficial use or misuse of TMFs is common and that clinical AI research is yet to benefit from the full value of TMF-research question alignment experienced by other areas of implementation research (18, 25, 86–89). Given the potential of unjustified heterogeneity to lower the accessibility of implementation research to relevant stakeholders, avoidance of TMF application may be preferable to their superficial use or misuse (6).

There are a number of tools which have been designed, validated and disseminated to reduce the underuse, misuse and superficial use of TMFs demonstrated here and in implementation research generally (2, 90). To aid researchers in the rationalised selection of TMFs, interactive open access libraries and selection tools are available with embedded learning resources (91, 92). Following selection of a TMF, many of the authors of more prominent TMFs develop and maintain toolkits to support the appropriate and effective mobilization of their TMF to varied applications (93, 94). There are also reporting guidelines and quality criteria which support peer reviewers and academic journal editors in identifying quality research and incentivizing researchers to adopt good practices. Apart from occasional exceptions in the present study however, none of these tools were mentioned or used (86, 89, 95, 96). The present study adds to these resources for implementation researchers working in clinical AI by summarizing TMF use to date within the field, with examples of good practice (55, 56, 85). Paradoxically, it seems that the limitation on improving TMF application is not the presence of solutions, but their implementation.

### 4.3. Strengths and limitations

A strength of this study is the eligibility criteria, which facilitated the large number of eligible articles relative to pre-existent bibliometric studies of TMF applications in implementation research (20–22). The study also summarizes TMF applications in clinical AI research, a prominent and growing category of digital health implementation research which had not yet been subject to any similar bibliometric studies. Without clear incentives for authors to report the perceived impact, mode or rationale of TMF application, a lack of information in eligible articles for the present study does not exclude a theoretical foundation. This risk of over-interpreting negative findings is not unique to the present study but is a further limitation to hold in mind (97). A final limitation comes from the eligibility criteria for the present study which focus on qualitative research of clinical AI, to maximise the representation of TMFs among eligible articles at the cost of implementation studies which exclusively use quantitative methods. Whilst this does limit comparability to bibliometric studies of guideline implementation research or other areas, it appears to have succeeded in identifying a greater sample of TMF applications within clinical AI than found by alternative criteria in more established fields of research (20, 21).

### 4.4. Future directions

Firstly, the ambiguity over the value of ensuring that implementation research that is “theoretically informed”, in a well-characterized and reproducible way, should be minimized through adequately resourced programmes of research. This is not in order to generate more TMFs, but to establish the impact of TMF application under current definitions of good practice. Without it, the challenge laid out in one of the first issues of the journal Implementation Science will continue to limit support from stakeholders influencing the implementation of TMFs: “Until there is empirical evidence that interventions designed using theories are generally superior in impact on behavior choice to interventions not so designed, the choice to use or not use formal theory in implementation research should remain a personal judgment” (19). A negative finding would also prevent future research waste in championing the proliferation and application of TMFs.

Secondly, if TMFs are proven to improve implementation outcomes then scalable impact within clinical AI and elsewhere cannot depend upon the oversight of implementation experts on any more than a small number of high priority implementation endeavors. Therefore, work to improve the accessibility and apparent value of existent TMFs and tools to promote their uptake should be prioritized (2, 91, 92). A focus on training and capacity building across a wider community of researchers and practitioners may also be beneficial (92, 98). Academic journal editors and grant administrators could be influential in endorsing or demanding relevant tools and guidelines, helping to improve the quality, consistency and transparency of theoretically informed clinical AI implementation research. Improved accessibility across existent TMFs would also help to tighten the relationship between frequency of application and efficacy of TMFs, helping to reduce the potentially overwhelming variety of TMFs available. If such a shortlist of popular TMFs emerged, with a clearer rationale and value for application, it could improve the accessibility of TMFs to a greater breadth of the implementation community. This could establish a virtuous cycle of improving frequency and quality of TMF application, mitigating against the researcher-practitioner divide described in implementation science (7).

## 5. Conclusion

Around a third of primary qualitative clinical AI research draws on a TMF, with no evidence of change in that rate. The selection of TMFs in these studies is extremely varied and often unaccompanied by any explicit rationale, which appears distinct from other areas of implementation research. In the context of the continual proliferation of TMFs and well-validated tools and guidelines to support their application, these data suggest that it is the implementation of interventions to support theoretically informed research, not their development, that limits clinical AI implementation research. Attempts to capture the full value of TMFs to expedite the translation of clinical AI interventions into practice should focus on promoting the rigor and frequency of their application.
