# Supplementary material for: Evaluating the translation of implementation science to clinical artificial intelligence: a bibliometric study of qualitative research
Source: Front Health Serv. 2023 Jul 10;3:1161822. doi: 10.3389/frhs.2023.1161822 (PMC10364639; doi:10.3389/frhs.2023.1161822)
Supplement: Supplementary file 1 [file Datasheet1.docx]

Search strategy across five databases – Executed 13^th^ October 2022

# MEDLINE (OVID)

1 (ethnological research or ethnograph* or life stor* or women* stor* or social construct* or postmodern* or post-structural* or post structural* or poststructural* or post modern* or post-modern* or feminis* or interpretative or interpretive action research or cooperative inquir* or co operative inquir* or co-operative inquir* or existential or unstructured or openended or open ended or life world or life-world or conversation analys?s or personal experience* or theoretical saturation or cluster sampl* or glaser* or participant observ* or human science or biographical method or heidegger* or colaizzi* or spiegelberg* or husserl* or foucault* or mixed method* or mixed-method*).ab,kf,kw,ti.

2 (corbin* adj2 strauss*).ab,kf,kw,ti.

3 (field adj (study or studies or research)).ab,kf,kw,ti.

4 (data adj1 saturat*).ab,kf,kw,ti.

5 ((discourse* or discurs*) adj1 analys?s).ab,kf,kw,ti.

6 (van adj (manen* or kaam*)).ab,kf,kw,ti.

7 (merleau adj ponty*).ab,kf,kw,ti.

8 ((interpretative or interpretive) adj (approach or research or data or method* or paradigm)).ab,kf,kw,ti.

9 (experiential adj (qualitative or knowledge or method*)).ab,kf,kw,ti.

10 ((lived or life) adj experience*).ab,kf,kw,ti.

11 ((theme* or thematic) adj1 (analys?s or data or synthesis or research)).ab,kf,kw,ti.

12 (account* adj1 (participant or patient* or clinician* or user* or professional* or carer* or family or stakeholder or open-ended or unstructured)).ab,kf,kw,ti.

13 (ethnonursing or phenomenol* or theoretical sampl* or observational method* or content analysis or emic or etic or hermeneutic* or semiotic*).af.

14 (narrative* adj (analys?s or synthes?s or data or research or methods or inquiry)).af.

15 (constant adj (comparative or comparison)).af.

16 (grounded adj (theor* or study or studies or research or analys?s)).af.

17 (purpos* adj sampl*).af.

18 (focus adj group*).af.

19 (qualitative adj1 (research or method* or data or study or studies or paradig* or analy*)).af.

20 Qualitative Research/ or Interview/ or Nursing Methodology Research/ or exp Diffusion of Innovation/

21 (Artificial intelligence or Boltzmann machine* or Long short-term memory or Gated recurrent unit or Rectified linear unit or Autoencoder or Backpropagation or Multilayer perceptron or Convnet or Support vector machine or Random forest or Lasso or Kernel or Elastic net* or Bayesian or Naive bayes or Genetic algorithm).ab,kf,kw,ti.

22 ((deep or convolutional or bayesian or neural or elastic) adj1 net*).ab,kf,kw,ti.

23 ((machine or deep or reinforcement or ensemble or convolutional) adj1 learning).ab,kf,kw,ti.

24 Big data/ or Decision support system, clinical/ or exp Algorithms/

25 ((algorithm* or computeri* or computer-based or computer based or machine-based or machine based or Computer assisted or Computer-assisted or Computer aided or Computer-aided or integrat* or technolog* or digital or electron*) adj3 (decision support or decision-support or decision aid or decision-aid)).ab,kf,kw,ti.

26 exp Health Occupations/ or exp Health Personnel/ or exp Persons/

27 (Perspective* adj1 (patient* or carer* or clinician* or doctor* or stakeholder* or nurse*)).ab,kf,kw,ti.

28 1 or 2 or 3 or 4 or 5 or 6 or 7 or 8 or 9 or 10 or 11 or 12 or 13 or 14 or 15 or 16 or 17 or 18 or 19 or 20 or 27

29 21 or 22 or 23 or 24 or 25

30 26 and 28 and 29

31 limit 30 to (humans and yr="2014 -Current")

# CINAHL (EBSCO)

MH ( Audiorecording or Interviews+ or “Grounded theory” or “Qualitative Studies” or “Research, Nursing” or “Focus Groups” or “Discourse Analysis” or “Content Analysis” or “Ethnographic Research” or “Ethnological Research” or “Ethnonursing Research” or “Constant Comparative Method” or Phenomenology or “Phenomenological Research” or “Implementation science” or “Usability study” ) OR TI ( Ethnonursing or ethnograph* or “life stor*” or “women’s stor*” or emic or etic or hermeneutic* or semiotic* or “participant observ*” or “social construct*” or postmodern* or post-structural* or “post structural*” or poststructural* or “post modern*” or post-modern* or feminis* or ((interpretative or interpretive) N1 (approach or research or data or method* or paradigm)) or “action research” or “cooperative inquir*” or “co operative inquir*” or “co-operative inquir*” or existential or experiential N1 (qualitative or knowledge or method*) or “human science” or “biographical method” or “theoretical sampl*” or glaser* or unstructured or open-ended or “open ended” or narrative* N1 (analys?s or synthes?s or data or research or method* or inquiry) or “life world” or life-world or “conversation analys?s” or “personal experience*” or “theoretical saturation” or “lived experience*” or “life experience*” or “cluster sampl*” or “observational method*” or “content analysis” or Heidegger* or Colaizzi* or Spiegelberg* or husserl* or Foucault* or van N1 manen* or van N1 kaam* or merleau N1 ponty* or Corbin* N2 strauss* or grounded N1 (theor* or study or studies or research or analys?s) strauss* N2 corbin* or data N1 saturat* or “field stud*” or “field research” or purpos* N1 sampl* or focus N1 group* or discourse* N1 analys?s or discurs* N1 analys?s or constant N1 comparative or constant N1 comparison or account* N1 (participant or patient* or clinician* or user* or professional* or carer* or family or stakeholder or open-ended or unstructured) paradigm* N1 qualitative (theme* or thematic) N1 (analys?s or data or synthesis or research) Perspective* N1 (patient* or carer* or clinician* or doctor* or stakeholder* or nurse*) or “mixed methods” or “mixed-methods” ) OR AB ( Ethnonursing or ethnograph* or “life stor*” or “women’s stor*” or emic or etic or hermeneutic* or semiotic* or “participant observ*” or “social construct*” or postmodern* or post-structural* or “post structural*” or poststructural* or “post modern*” or post-modern* or feminis* or ((interpretative or interpretive) N1 (approach or research or data or method* or paradigm)) or “action research” or “cooperative inquir*” or “co operative inquir*” or “co-operative inquir*” or existential or experiential N1 (qualitative or knowledge or method*) or “human science” or “biographical method” or “theoretical sampl*” or glaser* or unstructured or open-ended or “open ended” or narrative* N1 (analys?s or synthes?s or data or research or method* or inquiry) or “life world” or life-world or “conversation analys?s” or “personal experience*” or “theoretical saturation” or “lived experience*” or “life experience*” or “cluster sampl*” or “observational method*” or “content analysis” or Heidegger* or Colaizzi* or Spiegelberg* or husserl* or Foucault* or van N1 manen* or van N1 kaam* or merleau N1 ponty* or Corbin* N2 strauss* or grounded N1 (theor* or study or studies or research or analys?s) strauss* N2 corbin* or data N1 saturat* or “field stud*” or “field research” or purpos* N1 sampl* or focus N1 group* or discourse* N1 analys?s or discurs* N1 analys?s or constant N1 comparative or constant N1 comparison or account* N1 (participant or patient* or clinician* or user* or professional* or carer* or family or stakeholder or open-ended or unstructured) paradigm* N1 qualitative (theme* or thematic) N1 (analys?s or data or synthesis or research) Perspective* N1 (patient* or carer* or clinician* or doctor* or stakeholder* or nurse*) or “mixed methods” or “mixed-methods” )

AND

MH “Decision making, computer assisted+” OR TI ( “Artificial intelligence” or “Boltzmann machine*” or “Long short-term memory” or “Gated recurrent unit” or “Rectified linear unit” or Autoencoder or Backpropagation or “Multilayer perceptron” or Convnet or “Support vector machine” or “Random forest” or Lasso or Kernel or Elastic net* or Bayesian or “Naive bayes” or “Genetic algorithm” or or (deep or convolutional or bayesian or neural or elastic) N3 net* or (machine or deep or reinforcement or ensemble or convolutional) N1 learning or (algorithm* or computeri* or computer-based or “computer based” or machine-based or “machine based” or “Computer assisted” or Computer-assisted” or “Computer aided” or Computer-aided or technol* or digital or electron*) N3 (“decision support” or decision-support or “decision aid” or decision-aid) ) OR AB ( “Artificial intelligence” or “Boltzmann machine*” or “Long short-term memory” or “Gated recurrent unit” or “Rectified linear unit” or Autoencoder or Backpropagation or “Multilayer perceptron” or Convnet or “Support vector machine” or “Random forest” or Lasso or Kernel or Elastic net* or Bayesian or “Naive bayes” or “Genetic algorithm” or or (deep or convolutional or bayesian or neural or elastic) N3 net* or (machine or deep or reinforcement or ensemble or convolutional) N1 learning or (algorithm* or computeri* or computer-based or “computer based” or machine-based or “machine based” or “Computer assisted” or Computer-assisted” or “Computer aided” or Computer-aided or technol* or digital or electron*) N3 (“decision support” or decision-support or “decision aid” or decision-aid) )

AND

MH ( “Health personnel+” or Patients+ or Caregivers or Family+ or “Health Manpower+” ) OR SB ( Biomedical or Nursing or “Allied Health” or “Health Services Administration” or “Core nursing” )

# ACM Digital Library

(Title:(“Primary care” “Secondary care” “Tertiary care” Nurs* Carer* Caregiver* Health Healthcare Doctor* Nurse* Radiology Radiologist* Hospital* “General practice” Midwif* Surgery Surgeon* Ophthalm* Dermatol* Medic* Clinic* Pharma* Oncolog* Disease* “life sciences” Geriatri* Gerontol* Microbiolog* P*diatr* Rehabilitat* “social work” “social worker” “social workers” Psychiatry* Orthop* An*sthes* Patholog* Obstetric* Gyn*colog* Otorhinolaryngolog* Rheumatolog* H*matolog* Cardio* Audiolog* Urolog* Gastroenterolog* Physiotherap*)

OR

Abstract:(“Primary care” “Secondary care” “Tertiary care” Nurs* Carer* Caregiver* Health Healthcare Doctor* Nurse* Radiology Radiologist* Hospital* “General practice” Midwif* Surgery Surgeon* Ophthalm* Dermatol* Medic* Clinic* Pharma* Oncolog* Disease* “life sciences” Geriatri* Gerontol* Microbiolog* P*diatr* Rehabilitat* “social work” “social worker” “social workers” Psychiatry* Orthop* An*sthes* Patholog* Obstetric* Gyn*colog* Otorhinolaryngolog* Rheumatolog* H*matolog* Cardio* Audiolog* Urolog* Gastroenterolog* Physiotherap*))

AND

(Title:(“Artificial intelligence” “Boltzmann machine” “Long short-term memory” “Gated recurrent unit” “Rectified linear unit” Autoencoder Backpropagation “Multilayer perceptron” Convnet “Support vector machine” “Random forest” Lasso Kernel Bayesian “Naive bayes” “Genetic algorithm” “deep net” “deep network” “convolutional net” “convolutional network” “neural net” “neural network” “elastic net” “elastic network” “machine learning” “deep learning” “reinforcement learning” “ensemble learning” “convolutional learning” “computerised clinical decision support” “computerized clinical decision support” “computerised decision support” “computerized decision support”)

OR

Abstract:(“Artificial intelligence” “Boltzmann machine” “Long short-term memory” “Gated recurrent unit” “Rectified linear unit” Autoencoder Backpropagation “Multilayer perceptron” Convnet “Support vector machine” “Random forest” Lasso Kernel Bayesian “Naive bayes” “Genetic algorithm” “deep net” “deep network” “convolutional net” “convolutional network” “neural net” “neural network” “elastic net” “elastic network” “machine learning” “deep learning” “reinforcement learning” “ensemble learning” “convolutional learning” “computerised clinical decision support” “computerized clinical decision support” “computerised decision support” “computerized decision support”))

AND

(Title:(interview* thematic qualitative “nursing research methodology” Ethno* grounded “life story” hermeneutic semiotic “data saturation” “participant observation” “action research” “co-operative inquiry” existential “field study” “field studies” “field research” “biographical method” “Narrative inquiry” “Narrative analysis” “Narrative synthesis” “life world” “conversation analysis” “theoretical saturation” “lived experience” “life experience” “content analysis” “constant comparative” “discourse analysis” “discursive analysis” heidegger* “Implementation research” “Implementation study” “Usability study” “usability research”)

OR

Abstract:(interview* thematic qualitative “nursing research methodology” Ethno* grounded “life story” hermeneutic semiotic “data saturation” “participant observation” “action research” “co-operative inquiry” existential “field study” “field studies” “field research” “biographical method” “Narrative inquiry” “Narrative analysis” “Narrative synthesis” “life world” “conversation analysis” “theoretical saturation” “lived experience” “life experience” “content analysis” “constant comparative” “discourse analysis” “discursive analysis” heidegger* “Implementation research” “Implementation study” “Usability study” “usability research”))

# Scopus

( TITLE-ABS ( interview* OR ( ( theme OR thematic ) W/1 ( analys?s OR data OR synthesis OR research ) ) OR ( qualitative W/1 ( research OR method* OR data OR study OR studies OR paradig* OR analys* OR result* ) ) OR nursing-research-methodology OR ethnograph* OR ethnonursing OR ethnological-research OR grounded-theor* OR grounded-stud* OR grounded-research OR grounded-analys?s OR life-stor* OR women's-stor* OR emic OR etic OR hermeneutic OR semiotic OR data-saturat* OR participant-observ* OR postmodern* OR post-structural* OR feminis* OR ( ( interpretative OR interpretive ) W/1 ( approach OR research OR data OR method* OR paradigm ) ) OR action-research OR co-operative-inquir* OR existential OR ( experiential W/1 ( qualitative OR knowledge OR method* ) ) OR field-stud* OR field-research OR human-science OR biographical-method* OR theoretical-sampl* OR purposive-sampl* OR ( account* W/1 ( participant OR patient* OR clinician* OR user* OR professional* OR carer* OR family OR stakeholder OR open-ended OR unstructured ) ) OR ( narrative* W/1 ( analys?s OR synthes?s OR data OR research OR methods OR inquiry ) ) OR life-world OR conversation-analys?s OR theoretical-saturation OR lived-experience* OR life-experience* OR cluster-sampl* OR observational-method* OR content-analysis OR constant-comparative OR discourse-analys?s OR discurs*-analys?s OR heidegger* OR colaizzi* OR spiegelberg* OR van-manen* OR van-kaam* OR merleau-ponty* OR husserl* OR foucault* OR corbin* OR strauss* OR glaser* OR ( implementation W/0 ( science OR study OR research ) ) OR ( usability W/0 ( study OR research ) ) OR mixed-methods OR ( perspectiv* W/1 ( patien* OR care* OR clinicia* OR docto* OR stakeholde* OR nurs* ) ) ) OR AUTHKEY ( interview* OR ( ( theme OR thematic ) W/1 ( analys?s OR data OR synthesis OR research ) ) OR ( qualitative W/1 ( research OR method* OR data OR study OR studies OR paradig* OR analys* OR result* ) ) OR nursing-research-methodology OR ethnograph* OR ethnonursing OR ethnological-research OR grounded-theor* OR grounded-stud* OR grounded-research OR grounded-analys?s OR life-stor* OR women's-stor* OR emic OR etic OR hermeneutic OR semiotic OR data-saturat* OR participant-observ* OR postmodern* OR post-structural* OR feminis* OR ( ( interpretative OR interpretive ) W/1 ( approach OR research OR data OR method* OR paradigm ) ) OR action-research OR co-operative-inquir* OR existential OR ( experiential W/1 ( qualitative OR knowledge OR method* ) ) OR field-stud* OR field-research OR human-science OR biographical-method* OR theoretical-sampl* OR purposive-sampl* OR ( account* W/1 ( participant OR patient* OR clinician* OR user* OR professional* OR carer* OR family OR stakeholder OR open-ended OR unstructured ) ) OR ( narrative* W/1 ( analys?s OR synthes?s OR data OR research OR methods OR inquiry ) ) OR life-world OR conversation-analys?s OR theoretical-saturation OR lived-experience* OR life-experience* OR cluster-sampl* OR observational-method* OR content-analysis OR constant-comparative OR discourse-analys?s OR discurs*-analys?s OR heidegger* OR colaizzi* OR spiegelberg* OR van-manen* OR van-kaam* OR merleau-ponty* OR husserl* OR foucault* OR corbin* OR strauss* OR glaser* OR ( implementation W/0 ( science OR study OR research ) ) OR ( usability W/0 ( study OR research ) ) OR mixed-methods OR ( perspectiv* W/1 ( patien* OR care* OR clinicia* OR docto* OR stakeholde* OR nurs* ) ) ) )

AND

( TITLE-ABS ( artificial-intelligence OR boltzmann-machine* OR long-short-term-memory OR gated-recurrent-unit OR rectified-linear-unit OR autoencoder OR backpropagation OR multilayer-perceptron OR convnet OR support-vector-machine OR random-forest OR lasso OR kernel OR elastic-net* OR bayesian OR naïve-bayes OR genetic-algorithm OR ( ( deep OR convolutional OR bayesian OR neural OR elastic ) W/1 net* ) OR ( ( machine OR deep OR reinforcement OR ensemble OR convolutional ) W/1 learning ) OR ( ( algorithm* OR computeri* OR computer-based OR machine-based OR computer-assisted OR computer-aided OR technol* OR digital OR electron* ) W/3 ( decision-support OR decision-support OR decision-aid ) ) ) OR AUTHKEY ( artificial-intelligence OR boltzmann-machine* OR long-short-term-memory OR gated-recurrent-unit OR rectified-linear-unit OR autoencoder OR backpropagation OR multilayer-perceptron OR convnet OR support-vector-machine OR random-forest OR lasso OR kernel OR elastic-net* OR bayesian OR naïve-bayes OR genetic-algorithm OR ( ( deep OR convolutional OR bayesian OR neural OR elastic ) W/1 net* ) OR ( ( machine OR deep OR reinforcement OR ensemble OR convolutional ) W/1 learning ) OR ( ( algorithm* OR computeri* OR computer-based OR machine-based OR computer-assisted OR computer-aided OR technol* OR digital OR electron* ) W/3 ( decision-support OR decision-support OR decision-aid ) ) ) )

AND

( LIMIT-TO ( PUBYEAR , 2021 ) OR LIMIT-TO ( PUBYEAR , 2020 ) OR LIMIT-TO ( PUBYEAR , 2019 ) OR LIMIT-TO ( PUBYEAR , 2018 ) OR LIMIT-TO ( PUBYEAR , 2017 ) OR LIMIT-TO ( PUBYEAR , 2016 ) OR LIMIT-TO ( PUBYEAR , 2015 ) OR LIMIT-TO ( PUBYEAR , 2014 ) ) AND ( LIMIT-TO ( SUBJAREA , "MEDI" ) OR LIMIT-TO ( SUBJAREA , "HEAL" ) OR LIMIT-TO ( SUBJAREA , "NURS" ) OR LIMIT-TO ( SUBJAREA , "PHAR" ) OR LIMIT-TO ( SUBJAREA , "IMMU" ) OR LIMIT-TO ( SUBJAREA , "DENT" ) OR LIMIT-TO ( SUBJAREA , "Undefined" ) )

# Science Citation Index (Web of Science)

TS=(“Artificial intelligence” or “Boltzmann machine*” or “Long short-term memory” or “Gated recurrent unit” or “Rectified linear unit” or Autoencoder or Backpropagation or “Multilayer perceptron” or Convnet or “Support vector machine” or “Random forest” or Lasso or Kernel or “Elastic net*” or Bayesian or “Naive bayes” or “Genetic algorithm” or ((deep or convolutional or bayesian or neural or elastic) NEAR/1 net*) or ((machine or deep or reinforcement or ensemble or convolutional) NEAR/1 learning) or ((algorithm* or computeri* or computer-based or “computer based” or machine-based or “machine based” or “Computer assisted” or Computer-assisted or “Computer aided” or Computer-aided or technol* or digital or electron*) NEAR/3 (“decision support” or decision-support or “decision aid” or decision-aid)))

AND

TS=(interview* or ((theme or thematic) NEAR/1 (analys?s or data or synthesis or research)) or (qualitative NEAR/1 (research or method* or data or study or studies or paradig* or analys* or result*)) or “nursing research methodology” or ethnograph* or ethnonursing or “ethnological research” or “grounded theor*” or “grounded stud*” or “grounded research” or “grounded analys?s” or “life stor*” or “women’s stor*” or emic OR etic OR hermeneutic OR semiotic OR “data saturat*” OR “participant observ*” or postmodern* OR “post structural*” OR feminis* OR ((interpretative or interpretive) NEAR/1 (approach or research or data or method* or paradigm)) or “action research” OR “co-operative inquir*” or existential OR (experiential NEAR/1 (qualitative or knowledge or metho*)) OR “field stud*” OR “field research” or “human science” or “biographical method*” or “theoretical sampl*” or “purposive sampl*” or (account* NEAR/1 (participant or patient* or clinician* or user* or professional* or carer* or family or stakeholder or “open ended” or unstructured) or (narrative* NEAR/1 (analys?s or synthes?s or data or research or methods or inquiry)) OR “life world” OR “conversation analys?s” OR “theoretical saturation” or “lived experience*” OR “life experience*” or “cluster sampl*” or “observational metho*” or “content analysis” or “constant comparative” or “discourse analys?s” or “discurs* analys?s” or heidegger* or colaizzi* or spiegelberg* or “van manen*” or “van kaam*” or “merleau ponty*” or husserl* or foucault* or corbin* or strauss* or glaser* or (Implementation NEAR/0 (science or study or research)) or (Usability NEAR/0 (study or research)) or (Perspectiv* NEAR/1 (patien* or care* or clinicia* or docto* or stakeholde* or nurs*)) or Mixed-methods or “mixed methods”))

Results were then limited by ‘Research Areas’ to:

Medical Informatics OR Health Care Sciences Services OR Public, environmental, occupational health OR Neurosciences Neurology OR Psychiatry OR General Internal Medicine OR Radiology Nuclear Medicine Medical Imaging OR Pharmacology Pharmacy OR Mathematical Computational OR Biology OR Nursing OR Research Experimental Medicine OR Oncology OR Infectious Diseases OR Life Sciences Biomedicine and Other Topics OR Genetics Heredity OR Biotechnology/Applied OR Microbiology OR Rehabilitation OR Behavioural Sciences OR Surgery OR Substance Abuse OR Geriatrics Gerontology OR Pediatrics OR Microbiology OR Biomedical Social Sciences OR Parasitology OR Tropical Medicine OR Cardiovascular System Cardiology OR Endocrinology Metabolism OR Nutrition Dietetics OR Sport Sciences OR Toxicology OR Immunology OR Orthopedics OR Anaesthesiology OR Emergency Medicine OR Obstetric Gynecology OR Respiratory System OR Gastroenterology Hepatology OR Pathology OR Physiology OR Social Work OR Urology Nephrology OR Womens Studies OR Audiology Speech Language OR Dentistry Oral Surgery Medicine OR Ophthalmology OR Otorhinolaryngology OR Anatomy Morphology OR Integrative Complementary Medicine OR Rheumatology OR Virology OR Allergy OR Dermatology OR Family Studies OR Medical Laboratory Technology OR Mycology OR Transplantation OR Hematology OR Reproductive Biology
